# Challenges to diabetes self-management for adults with type 2 diabetes in low-resource settings in Mexico City: a qualitative descriptive study

**DOI:** 10.1186/s12939-019-1035-x

**Published:** 2019-08-23

**Authors:** Robin Whittemore, Mireya Vilar-Compte, Selene De La Cerda, Denise Marron, Rosabelle Conover, Roberta Delvy, Annel Lozano-Marrufo, Rafael Pérez-Escamilla

**Affiliations:** 1 Yale School of Nursing, 400 West Campus Drive, West Haven, CT 06516 USA; 2 Universidad Iberoamericana, Prolongación Paseo de Reforma 880, Lomas de Santa Fé, 01219 Álvaro Obregón, Mexico City Mexico; 3 Yale School of Public Health, 60 College Street, New Haven, CT 06510 USA

**Keywords:** Type 2 diabetes, Diabetes self-management in Mexico, Health disparities

## Abstract

**Background:**

The prevalence of type 2 diabetes (T2D) in Mexico is one of the highest in the world, with high morbidity and mortality, and difficulty meeting glycemic targets. The purpose of this study was to identify the challenges for T2D self-management as perceived by both adults with T2D and health care providers in primary health clinics from *Seguro Popular* in Mexico City.

**Methods:**

This was a qualitative descriptive study conducted in three *Seguro Popular* primary care clinics in Mexico City using convenience sampling. Semi-structured interviews were conducted with participants and data were analyzed using a content analysis approach.

**Results:**

The sample included 20 adults with T2D [52.5 years old (SD = 9.9), diagnosed with T2D for 12.3 years (SD = 6.3), mean A1C of 9.8% (SD = 2.4), 80% female, 90% with financial insecurity] and 19 providers [primarily female (78.9%), mean age of 41.6 years old (SD = 11.4), 12.3 mean years in practice (SD = 8.50)]. Personal challenges included cultural beliefs, lack of resources, challenges to lifestyle modification, lack of family support/competing demands, and mental health issues. System level challenges included lack of resources, perceived quality of care, and patient engagement barriers.

**Conclusions:**

Evidence-based diabetes self-management programs need to become more accessible, taking into consideration the social determinants of health and building upon current initiatives to improve early diagnosis and treatment of T2D. Cultural beliefs, personal control, and low health literacy influence diabetes self-management in adults with T2D with limited resources. Mental health and financial challenges of adults with T2D will require multidisciplinary team-based care. Future research on best practices to implement and scale-up evidence-based patient-centered T2D prevention and DSME programs for the poor and underserved is warranted in Mexico and world-wide.

## Background

Type 2 diabetes (T2D) is a worldwide public health epidemic with persons in low- and middle-income countries affected disproportionately [1, 2]. According to a 2017 report by the International Diabetes Association, 79% of the global diabetes population resided in low- and middle-income countries, where resources to treat diabetes and its complications might be less available than in high-income countries [3].

Mexico is at the forefront of the epidemic. The 2016 Mexican Health System Report from the Organization of Economic Cooperation and Development (OECD), highlighted that diabetes prevalence in Mexico reached 15.9%, more than double the OECD average of 6.9% [4]. In Mexico City, a recent study from the Mexican Institute of Public Health [5], revealed that 13.9% of the adult population had T2D. Mortality associated with T2D in Mexico continues to increase; between 1998 and 2008 mortality from T2D doubled [5].

As in other countries, T2D has important economic implications in Mexico. According to Barquera and colleagues [6], around 15% of the total health expenditures in Mexico are related to diabetes care. The authors estimated direct costs of T2D (2006: outpatient USD$ 717,764,78, inpatient USD$ 223,581,099) and indirect costs (2005, USD$ 177,220,390) that reflect the diabetes-related crisis in Mexico. Moreover, it has also been documented that households with at least one member diagnosed with diabetes, hypertension or both, reported health expenditures that were 25–34% significantly higher than households without such individuals [7].

Among those with diagnosed T2D in 2016, 87% reported following a medical treatment; however only 16.6% in the social security health care system and 22.3% in the public health insurance system (i.e. *Seguro Popular*) reported meeting targets for glycemic control [8]. This reveals important challenges in clinical and self-management practices that need to be addressed in adults with T2D in Mexico.

Adults with T2D in Mexico often lack knowledge and understanding about T2D etiology and self-management. Many Mexican adults do not associate diet and physical activity with T2D, instead they believe that the primary etiology of T2D is extreme fright, shock, or a sudden scare [9–11]. T2D self-management is sub-optimal with 12% of adults with T2D not following self-management at all (blood glucose monitoring, dietary modifications, physical activity), 60% addressing one or two behaviors, and only 28% performing all self-management behaviors [10]. Indeed, very few adults with T2D in Mexico understand the importance of dietary modification or physical activity as part of diabetes self-management (24 and 2% respectively) [5]. Furthermore, low health literacy, high use of herbal remedies, financial barriers, difficulty following dietary recommendations, depressive symptoms, and limited communication with providers have also been reported as risk factors for poor diabetes self-management [12, 13].

The purpose of this study was to identify the challenges for T2D self-management as perceived by both adults with T2D and health care providers in primary health clinics from *Seguro Popular* in Mexico City.

## Method

This was a qualitative descriptive study [14] conducted in three *Seguro Popular* primary care clinics in two counties in Mexico City using convenience sampling. Qualitative description is a ‘method of naturalistic inquiry that uses low inference interpretation to present results in everyday language.’ It allows for exploration of health problems of vulnerable populations who have complex cultural and clinical factors influencing health outcomes and their interaction with the health care system. Understanding these factors from the perspective of persons living with a specific condition can improve intervention development and clinical care [15].

Individuals who obtain health care at these clinics are covered by public health insurance. This public insurance offers universal access to health services that include: medical visits, laboratory testing, free medicines and referrals to highly specialized health centers if it is needed. In 2016, approximately 50% of the population who reported affiliation to a health care system were covered by *Seguro Popular* [16]. By 2018, *Seguro Popular* provided health care to over 53 million people nationwide [17]. Adults enrolled in the *Seguro Popular* system have a mean age of 40.6 years, with 64% male, 12% reporting a diagnosis of T2D, and the majority with low income (97%). In the total adult population in Mexico City, individuals have a mean age of 43.6 years, 53% male, 13% reporting a diagnosis of T2D, and 55% with low income. Seventy-nine percent of adults enrolled in *Seguro Popular* have at least a sixth-grade education level [18].Thus, adults in *Seguro Popular* are socio-economically vulnerable.

Appropriate Institutional Review Board approval was obtained from Yale University and the Center for Welfare Analysis and Measurement (CAMBS for its acronym in Spanish). Adults with T2D were eligible for the study if they were between 21 and 65 years of age and had been diagnosed with T2D for at least one year. Providers were eligible if they had at least 3 months of experience caring for patients with T2D at a *Seguro Popular* clinic. A coordinating official of the Mexico City’s Health Department introduced the research team to administrators at three *Seguro* Popular clinics based on the fact that they had a sufficient number of adults with type 2 diabetes with varying metabolic control. Permission was obtained from clinic directors to conduct research at the clinic. Research team members did not have a previous relationship with administrators, health care professionals, or patients at any of the clinics. Flyers were distributed to the clinic settings and eligible adults with T2D and providers were provided information about the study by a trained research assistant. If interested, an appointment was scheduled for informed consent and the interview. Twenty-two providers were invited to participate, with 3 declining (86% participation rate). Thirty-five adults with T2D were invited to participate, with 15 declining participation in the study (57% participation rate). The most frequent reason for declining participation was lack of time.

Semi-structured interviews were conducted with adults with T2D to understand their experiences living with T2D and with providers on their experiences caring for adults with T2D by trained research assistants. Interview questions were collaboratively developed by a multidisciplinary team with expertise in nursing, public health, health services, and the Mexico health care system. Our overarching aim was to assess individual, social, and health care system challenges and inequities as identified in the social ecological model [19]. (Tables 1 and 2). Interviews were conducted in a private location at the clinic and averaged 27 min for adults with T2D and 37 min for health care providers. All interviews were audio-recorded, conducted in Spanish and transcribed verbatim by trained bilingual research assistants. Field notes were not recorded. Participants also completed a brief demographic questionnaire. All participants were assigned a code number and names were not transcribed in any interviews to assure confidentiality. Participants continued to be recruited until information saturation was achieved. Participants with T2D received 100 pesos for their time and providers received a book on physical activity and dietary guidelines for the Mexican population.

**Table 1:** Interview Guide: Adults with T2D

Interview Questions	
What you believe are the causes of T2D?	
What kinds of things do you do to help manage your T2D?	
What kinds of healthcare do you receive for your T2D? How often?	
How is your family involved with your T2D care?	
What challenges do you face living with T2D? What is hard about having diabetes?	
What kind of information would help you take good care of your health/T2D?	
What do you think about the idea of using text messages to manage your healthcare?	
How could we use text messaging to help you take good care of your health/T2D?	
Would any of you like to add any other comments?

**Table 2:** Interview Guide: Health Care Providers

Interview Questions	
What are your professional duties as a health care provider?	
What kind of care do adults with T2D receive at this clinic?	
What are the cultural barriers you face in caring for adults with T2D?	
What are the challenges you face in caring for adults with T2D?	
What are some examples of cases of successful management of T2D?	
What is the importance of education in the care of adults with T2D? What are the key elements of such education?	
What are the barriers and facilitators to promoting glucose self-monitoring in adults with T2D?	

Data were analyzed using a content analysis approach [20]. To assure methodological rigor, two bilingual research team members independently coded the 39 interviews (RC and DM). A coding team of the two coders (RC and DM), the primary investigator (RW), and another research assistant (RD) met frequently during the coding process to review the coding process and resolve discrepancies through discussion. The primary investigator had expertise in qualitative methodology.

After the first five interviews were coded by both coders using line by line coding, the research team met and developed a code book based on 15 coding categories. Coders then re-coded the initial 5 transcripts using the code book to assure that the code book was complete and agreement on coding was achieved by the two coders. Upon completion of coding all transcripts in Spanish, codes, sub-categories within codes, and exemplar quotes were translated into English. Codes and sub-categories were then collapsed into an overarching conceptualization of themes and subthemes by the coding team, using Microsoft Word and Microsoft Excel. We identified 8 themes (5 personal challenges shared by providers and adults with T2D, 1 system-level theme endorsed by providers and adults with T2D, 1 system-level theme endorsed by providers, and 1 endorsed by adults with T2D). To ensure methodological rigor, we re-examined data to assure that the coding process continued to be reflective of the participant’s perspective as codes were collapsed into themes of higher abstraction. We maintained an audit trail of all coding decisions throughout the process and used a consensus-making process within our diverse interdisciplinary team for all aspects of study design, implementation, and data analysis, to assure that our methods were rigorous and our final conceptualization of the data was reflective of the participants’ perspective [21]. The Consolidated Criteria for Reporting Qualitative Research was used to guide the reporting of this study [22].

## Results

Twenty adults with T2D completed interviews. They were on average 52.5 years old (SD = 9.9), formally diagnosed with T2D for 12.3 years (SD = 6.3), had a mean glycosylated hemoglobin (A1C) of 9.8% (SD = 2.4) indicating poor metabolic control, and 80% were female. Eighty percent were married, 45% currently working, and 55% reported preschool or primary school as their highest level of education. The majority of adults with T2D (70%) reported that they had enough money just to meet basic needs, 20% reported that they did not have enough money, and only 10% reported that they had enough money to live comfortably. The majority of the sample reported other comorbidities, including hypertension (60%), high blood cholesterol (55%), depressive symptoms (50%), gastritis or ulcer (50%), heart disease (30%), and kidney disease (15%). Sixty-five percent of the sample had 3 or more comorbidities. When asked to self-report their health status, 30% rated their health as good or very good, 30% as ‘more or less okay’, and 40% rated their health as bad or very bad. Our sample was representative of adults with limited resources who access health care at *Seguro Popular* clinics [18].

Nineteen providers, including 7 general practitioner physicians, 5 nurses, 3 social workers, and 1 nutritionist, psychologist, dental surgeon, and physical activity counselor completed interviews. Providers were primarily female (78.9% which is reflective of health providers in Mexico at approximately 73% female), had a mean age of 41.6 years old (SD = 11.4), 12.3 mean years in practice (SD = 8.5), and 9.2 years working in their current clinic (SD = 10.0).

Adults with T2D and providers identified common challenges to T2D self-management, which were categorized into personal and system level challenges. Personal challenges included: 1) cultural beliefs surrounding T2D causation and treatment; 2) lack of resources to access healthy food, medications, and/or supplies for diabetes management (i.e. glucometer, glucose test strips, lancets) 3) challenges to lifestyle modification due to low health literacy, difficulty in changing established habits, and perceived lack of symptoms; 4) lack of family support for lifestyle changes or competing demands with work and family; and 5) mental health issues. There were several system-level challenges to T2D management that were organized into three themes: 1) clinic resources (identified by patients and providers); 2) perceived quality of care (identified by adults with T2D); and 3) patient engagement barriers (identified by providers). Themes and exemplars of coding statements are provided in Tables 3 and 4.

**Table 3:** Personal Challenges: Themes and Exemplar Coded Statements

Personal Challenge Theme	Adults with T2D	Provider
Cultural beliefs	Believe medicine will fix diabetes Believe stress causes diabetes Believe insulin causes blindness Believe that diabetes is not something controllable	Patients believe T2D caused by ‘susto’ Patients believe home remedies will cure diabetes Patients believe insulin doesn’t work. Causes blindness Patients relate diabetes to death
Lack of resources	Not enough money to buy vegetables Have to buy different food from family Not enough money for diabetes supplies Lack funds to purchase adequate food	Unable to see nutritionist due to cost Eat many tortillas as they are inexpensive Patient can’t afford syringes/glucometer strips Challenges of living in poverty
Lifestyle change	Unable to understand educational materials Have a hard time giving up favorite foods Like feeling full and diabetes diet isn’t satisfying Work schedule makes it hard to eat healthy Forget to take medication Follow diabetes care only when feeling sick	Unable to read/write Difficult to stop drinking soda Misinformation/lack of information about diabetes Lots of events with opportunity to eat poorly Many work and eat food from street vendors Prioritize other activities
Lack of family support	Make foods to please family not self Family request unhealthy food at the store Family does not support lifestyle change Family too busy to help with diabetes care	Lack of family support to help with health Machismo can be issue for some families Many patients abandoned by family Don’t have family support for lifestyle change
Mental health	Feels depressed/sees life as ending Believe diagnosis was a death sentence No longer feels like same person – impotent Feels hopeless	Depression makes adherence/attendance difficult Many barriers to psychological care Considerable stigma to psychological care Patients not aware of value they have as person, in family, and community

**Table 4:** System Challenges: Themes and Exemplar Coded Statements

System Challenge Theme	Adults with T2D	Provider
Clinic resources and quality of care	No medication/insulin available Only able to obtain some medications at the clinic No glucometer or strips available	Lack of staff time (e.g. rushed appointments) Lack of staff availability Difficult to build rapport with patients Constant medication/supplies shortage
Staff insensitivity	Perceive that provider is critical and rude if having difficulty with diabetes management Does not feel listened to by provider Perceives that provider scares them about complications	
Patient Engagement Barriers		Patients move frequently Patients miss or forget appointments Patients live far away – difficult transportation Staff turnover prevents patients from coming to clinic

### Personal challenges

#### Cultural beliefs

Adults with T2D and providers reported many cultural beliefs about T2D causation and treatment. While some adults with T2D understood that genetics and lifestyle contributed to the development of T2D, others lacked understanding of these contributory factors. As one adult with T2D stated, *“The truth is I don’t know why I got diabetes. People say because of [a sudden] fright, or lack of nutrition, but the truth, I don’t know.”* (Female, age 36). Several adults with T2D had misconceptions about T2D medications, with strong fears expressed for insulin. One adult with T2D stated, “*Too much medication makes you sick. The pills themselves turn into rocks in your kidneys, so if the doctor tells me to take three pills, I take two.”* (Female, age 52). Providers and adults with T2D reported the misconception that insulin leads to blindness or amputation. As one provider stated, *“The patient often brings myths or legends about insulin because of their personal experiences, because they have family members who have become blind, or have lost some extremity.”* (Female, MD). Adults with T2D and providers also spoke about the use of herbs or other remedies used to lower blood glucose heard about through family and friends (e.g. seeds, vegetable peel). Lastly, providers also spoke about the culture of health care seeking behaviors in Mexico, a culture of treatment rather than a culture of prevention that influences how adults with T2D may or may not access the health care system. One provider stated, *“In our culture, we don’t present to the health sector until we are sick. We do not have…a culture of prevention. We go to the doctor when something already hurts us, no? And culturally we have been taught that the longer we tolerate pain or the longer we tolerate symptoms, the stronger we are.”* (Female, Social Worker).

#### Lack of resources

Lack of access to resources interfered with optimal diabetes management. Specifically, lack of access to healthy food, medications, and supplies, such as glucometer strips was also expressed by adults with T2D and providers as a challenge to T2D management. Healthy eating for T2D was perceived as expensive and therefore, not a priority in families with limited income. As one provider stated, *“…when one indicates to them to follow nutritional therapy, they [adults with T2D] state that they do not have enough money to…buy the foods that are indicated.”* (Male, MD). An adult with T2D stated, *“There is no point for me to see the nutritionist, for them to tell me what to eat if I cannot buy it.”* (Female, age 40) One provider reported that *“patients will eat up to 20 tortillas in one meal because they can’t afford anything else.”* (Male, MD). Similar challenges were reported with respect to medications (if they had to pay for them) and blood glucose monitoring supplies. One provider shared a recent interaction they had with a patient with T2D – *“It’s that ‘Doctor, the strips cost me 500 pesos for 50 strips…Do I eat or do I buy the strips? Pay the rent or buy my strips?’”.* (Female, MD).

#### Lifestyle modification

Challenges to lifestyle modification due to low health literacy, difficulty in changing established habits, and perceived lack of symptoms were also reported. Providers spoke of the challenges of providing information on diabetes management to adults with T2D with limited education and health literacy. As one provider stated, *“Many completed no more than some years of elementary school, others know how to read, or half know how to write, but in reality they did not complete any type of basic education.”* (Female, MD). One adult with T2D stated, *“They remind me of the things I can and cannot eat, I like that. They also give a big paper with that but I don’t know how to read, so I always leave it.”* (Female, age 64). Changing established habits to eat healthy or exercise were also reported as difficult. One provider stated, *“The majority of patients are already over 60 years, so, to suddenly change nutritional habits is very complicated for them.”* (Female, Social Worker). One adult with T2D stated, *“[The doctor] tells me to take care of my nutrition. Don’t drink soda and try to have a well-balanced diet. But…I cannot do that. If I could I would because it’s for my own good but even if I wanted to, I couldn’t do it.” (*Female, age 40). Unhealthy ‘street’ food, frequent ‘carnivals’ with many opportunities to eat poorly, and cultural preferences [atole (corn flour-based beverage), pan dulce (sweet bread), and tortillas] also were expressed as barriers to healthy eating. Adults with T2D also expressed that co-morbidity and complications from T2D could make diabetes self-management tasks difficult (e.g. eyesight, pain in feet). In addition, lack of symptoms interfered with establishing positive health behaviors. As one provider stated, *“Since they do not have any manifestations (of illness), they consider that they do not need any management.”* (Female, Nurse). An adult with T2D stated, *“I take care of myself when I feel bad, then yes, it’s vegetables and no soda, no coffee, but when I feel good again, I return to the same old thing.”* (Female, age 47).

#### Lack of family support

Adults with T2D and providers also perceived that a lack of family support as well as competing demands with work and family contributed to challenges for adults with T2D, particularly women, to follow treatment recommendations, including attending clinic appointments. One provider stated, *“The women of the house who have diabetes…they cannot do the nutrition…because of considerations of how the other members of the family are going to eat what they are accustomed to eat…it ruins the initiative that they have [decreases motivation].”* (Female, Psychologist). Another provider stated, *“There are also adults who very often are abandoned…really they have children, but…no one is in charge of taking care of them.”* (Female, Nurse). A woman with T2D stated, *“I have 18 years with diabetes and [my doctor] would ask me why I couldn’t control it. I would tell her I have to take care of my family…they ask me to relax, to exercise, but I cannot. I don’t have time.”* (Female, age 57).

#### Mental health

The last personal challenge to T2D management expressed by providers and adults with T2D was mental health issues, particularly depressive symptoms. For some adults with T2D, their depressive symptoms were related to their perception that T2D would contribute to their death. As a result of personal experiences, they lacked awareness that effective T2D management could prevent complications and improve the quality of their lives. Providers reported that patients often related diabetes to death. As one adult with T2D stated, *“I got depressed. I wanted to kill myself, ‘What do I have to live for anymore if I have this very bad disease’. I couldn’t see good anymore.”* (Male, age 57) Another adult with T2D stated, *“I felt like I was going to die. All my family has died as consequence of [diabetes] and very young…before they were 50 years old. My dad among them.”* (Female, age 36).

### Health care system-level challenges

#### Clinic resources and quality of care

There were several system level challenges to T2D management. Providers and adults with T2D identified that clinic resources and perceived quality of care contributed to difficulties with T2D management. One provider stated, *“Almost always we are missing the A1C [equipment to measure blood glucose], or if we have cholesterol, sometimes we do not have triglycerides, or sometimes we do not have this, microalbumin [test].”* (Female, MD). Another provider stated, “*When there is no medication, many patients get bothered and, for example, they tell us, ‘What is [the clinic] good for…if it is not going to give medication?”* (Female, Nurse). Lack of resources and time were sources of stress for providers. They reported that there was lack of personnel to complete tasks (e.g. registered nurses), lack of time to meet the needs of patients, as well as inconsistent supplies of medications for patients.

#### Staff insensitivity

Some adults with T2D reported that perceived staff insensitivity to their illnesses and concerns contributed to distress and discouragement. One adult with T2D stated, *“He [doctor] tells me I don’t try, that I don’t want to control my sugar.”* (Female, age 50). Another stated, *“Sometimes I don’t like it. I know I am going to die but he [my doctor] is rude sometimes. I don’t like that and I get very depressed.”* (Female, age 36).

#### Patient engagement barriers

Lastly, providers identified that there were barriers to patients’ consistent engagement in clinical care. As one provider stated, *“Many people from here come to rent, so suddenly they disappear and reappear in 3 or 4 years.”* (Female, MD). Other providers stated that patients miss or forget appointments related to regular clinic visits, follow-up laboratory tests, or clinical consultants for various reasons such as time, distance, or change in clinical staff.

## Discussion

Through our qualitative study, interviewing adults with T2D and providers, we identified numerous challenges related T2D care in Mexico that are important to contextualize within the current policy trends in Mexico. In the last decade, several policy frameworks and interventions related to T2D care have been implemented; among them the National Strategy for the Prevention and Control of Overweight, Obesity and Diabetes and the expansion of the *Seguro Popular* system [23, 24]. This has contributed to a greater emphasis of diabetes in the national policy agenda, leading to an increase in screening for T2D, the establishment of diabetes care centers [25], and the implementation of public policies such as sugar-sweetened beverages taxation [26]. However, there is mixed evidence about the impacts of such initiatives and policies. For example, prior research has found that implementing sugar-sweetened beverage taxes has been associated with a decrease in sugar-sweetened beverages consumption among the general population [27], and access to T2D care services among population insured in *Seguro Popular* has improved [28]. However, there are several studies that demonstrate that such policies have not improved T2D treatment effectiveness, adherence to lifestyle modifications related to diet and physical activity [29, 30], and health behaviors such as blood glucose testing [28]. Hence, outcomes in adults with T2D remain poor [23, 29];and there has not been a significant decrease in T2D related complications and mortality [8, 19, 23, 31]. These challenges are recognized worldwide, with increasing prevalence of T2D and associated complications.

Similarly, in other studies, poor knowledge about diabetes among adults with T2D have been reported [32] and limited self-efficacy for meeting targets for glycemic control [33, 34]. Diabetes education has not been a systematic diabetes care strategy in the public health sector in Mexico, and in *Seguro Popular* clinics these types of education programs are not provided. In the best-case scenario, some diabetes support groups have been implemented such as peer-support groups (*Grupos de Ayuda Mutua*, in Spanish) in Mexico City’s local public health system [33].

Hence, the findings of our qualitative research mirror such challenges in accessing quality services for adults with T2D of limited resources and insured in *Seguro Popular*. Findings indicate that it is important to: 1) improve access to diabetes self-management education (DSME); 2) address myths and misconceptions of adults with T2D; 3) address challenges of healthy eating with limited resources; 4) engage families and communities in T2D prevention and management; 5) address mental health needs; and 6) promote continuity of care with an emphasis on the provider-patient relationship and access to specialized care.

DSME is necessary for all people with T2D as well as those at risk for developing T2D. Considerable evidence supports the benefit of DSME on clinical, behavioral, and psychosocial outcomes [35]. DSME consists of individual and group-sessions to enhance knowledge, skills, and problem-solving of diabetes self-management. Standards of care in the United States have been developed with core content areas identified – diabetes pathophysiology and treatment options, healthy eating, physical activity, medication usage, self-monitoring, preventing and treating acute and chronic complications, healthy coping, and problem solving [36]. Expansion of the group-based programs for T2D in clinics that care for the poor and underserved globally is warranted. In addition, improving access to blood glucose monitoring equipment and supplies is needed, particularly for adults with T2D who are learning diabetes self-management skills or those treated with insulin [37].

DSME that incorporates empowerment based-support may be particularly relevant to adults with T2D with low health literacy, as this approach facilitates the development of personal responsibility and control of daily T2D decisions through personalized goal setting, problem solving, and social/emotional support [38]. Empowerment-based DSME programs have demonstrated positive findings in Latinos living in the United States [39, 40]. Empowerment-based approaches are also relevant for those with low health literacy due to the focus on the needs of the person with T2D and the facilitation of personalized problem-solving [41]. Additional strategies to enhance health literacy for adults with T2D include simplifying educational content to a few key points, using lay language and pictures, and confirming understanding through interactive activities [42].

While it is well-established that there are numerous cultural beliefs on the causation, progression, and management of T2D that interfere with optimal diabetes self-management, greater efforts to dispel myths and misconceptions are needed. Consistent with our findings, previous studies have reported cultural beliefs in Mexican adults with diabetes, including that diabetes can be caused by a scare or a stressful situation (*coraje*, in Spanish) and that medication is the reason for complications, not diabetes itself. Those studies also identified fear toward insulin use, providing strong support that these beliefs are deeply rooted among Mexicans [9, 43]. Intrinsic motivation to establish healthy behaviors for T2D is difficult when adults with T2D believe that they do not have any control in improving their health trajectory; this belief has been identified as fatalism and may be related to religious beliefs or economic struggles [44]. Continued efforts to screen adults at risk for T2D whenever they engage in the health care system to initiate treatment at diagnosis are greatly needed to prevent long-term complications of blindness, end-stage kidney disease, and cardiovascular disease. Efforts to assess T2D risk and implement T2D prevention strategies are also indicated, as considerable evidence on the effectiveness of T2D prevention programs has been demonstrated globally [45].

As evidenced in this study, health care providers and adults with T2D agree that economic struggles often result in limited funds for healthy food. This has been reported in prior studies which highlight the barriers to healthy eating among vulnerable populations [11, 46, 47], which compromises T2D self-management. For example, in a qualitative study with low-income adults with T2D in one urban area in a southern Mexican state, the perception of healthy food being expensive and not satiating was reported [12]. Thus, DSME for adults with T2D with low socioeconomic status will also need to include strategies for healthy eating when resources are limited. Traditional Latin American cooking that involves flavoring foods with vegetables and spices has rapidly been replaced by inexpensive, highly processed, high caloric foods, including sugar-sweetened beverages. In the past 15 years, sugar-sweetened beverage consumption has increased over 157% in Mexico to approximately 434 l per capita per year, the highest in the world [48]. Caloric intake has increased by approximately 650 kcal/person/day, with increases in sugar, animal source foods, and vegetable oil consumption with decreases in cereal and legume intake [49]. Strategies to address these alarming trends need to be developed, disseminated, and evaluated not only in Mexico, but globally. Policies to limit sugar-sweetened beverages and DSME programs are one starting point for these efforts. Engaging families and communities in T2D prevention and treatment is also indicated. Family-based programs for adults with T2D can improve self-efficacy, perceived social support, and diabetes self-management [50, 51]. In addition, encouraging family members, who may also be at risk for T2D, to make lifestyle changes of healthy eating, physical activity, and weight loss/maintenance may also decrease their personal risk for T2D.

Addressing the mental health needs of adults with T2D is also indicated. Providers and adults with T2D reported that depressive symptoms interfered with diabetes self-management and quality of life. The prevalence of depression and anxiety are much higher in adults with T2D than in the national population. Among the general population in Mexico, the prevalence of depression is 9.1% and the prevalence of anxiety disorders is 14.3% [52]. In contrast, the prevalence of depression in adults with T2D ranges from 46 to 63% and the prevalence of anxiety is approximately 55%, which are staggering statistics [53]. While these estimates are based on self-report of depressive and anxiety symptoms, they do draw attention to this significant health problem. Annual screening for increased depressive symptoms has been recommended by the American Diabetes Association and is currently a standard of care in the United States [54]. Assessment and treatment of these disorders within the clinical setting in clinics with limited resources will require additional training of health care providers and more adjunct health professionals (such as registered nurses, social workers, and psychologists) to meet the needs of the population. Thus, enhancing the health care system within clinics and strategizing cost-effective coordination of multidisciplinary care is an important future priority. Utilizing well-trained community health workers to extend the work of health care providers is a potentially cost-effective strategy to improve outcomes in low-resource settings [39].

As with many countries world-wide, health care initiatives in Mexico focus on the medical management of chronic conditions, with less attention on the individual, family, and health care system factors that influence the patient experience and health outcomes [4]. Future research on the implementation of evidence-based screening, treatment, and prevention of T2D programs for vulnerable populations globally, is critically important moving forward. Through implementation science, the strengths of a community or health care system are considered while addressing challenges, thus enhancing scale-up of new programs [55]. Community-based participatory research, is increasingly recognized as critical to the successful translation of current evidence into sustainable and effective programs that build upon contextual knowledge and practice [56].

There were several limitations of this study. While we interviewed adults with T2D and providers at 3 *Seguro Popular* clinics, they were all in one general geographical location in Mexico City although it mirrors many of the inequalities in income and access to health care services faced in the country. Adults with T2D were primarily female, married, and living with T2D for over 10 years. Understanding the complex cultural and contextual factors influencing health outcomes provides potential target for assessment and intervention in other vulnerable populations with T2D or at risk for T2D.

## Conclusions

In summary, adults with T2D and providers in *Seguro Popular* clinics identified numerous challenges to managing T2D. Evidence-based DSME programs need to become more accessible, considering the social determinants of health and building upon current initiatives to improve prevention, early diagnosis and treatment of T2D globally. Cultural beliefs, personal control, and low health literacy need to be addressed in DSME programs. Mental health and financial challenges of adults with T2D will require multidisciplinary team-based care. Future research on best practices to implement and scale-up evidence-based patient-centered T2D prevention and DSME programs for the poor and underserved is warranted in Mexico and world-wide. Findings from this study have major implications for the Latin American and Caribbean Region given the sharp increase in the proportion of low income families living in urban areas over the past decades [57, 58].

## Data Availability

Datasets used and analyzed during this study are available from the corresponding author on reasonable request.

## Abbreviations

OECD: Organization of economic cooperation and development
SD: Standard deviation
T2D: Type 2 diabetes
